# Effects of preoperative carbohydrate loading on postoperative outcomes in patients undergoing laparoscopic cholecystectomy: a systematic review and meta-analysis

**DOI:** 10.1097/MS9.0000000000003417

**Published:** 2025-05-26

**Authors:** Muhammad Saeed Qazi, Muhammad Omar Larik, Hamza Irfan, Maimoona Khan, Komal Zulfiqar, Shree Rath, Mian Iqbal Ahmed Sheikh, Hind A. M. Elamin, Javed Iqbal, Muhammad Usman, Pratik Bhattarai

**Affiliations:** a Department of Medicine, Medical Research Centre, Liaquat University of Medical and Health Sciences, Jamshoro, Pakistan; b Department of Medicine, Dow International Medical College, Karachi, Pakistan; c Department of Medicine, Shaikh Khalifa Bin Zayed Al Nahyan Medical and Dental College, Lahore, Pakistan; d Department of Surgery, Dow University of Medical and Health Sciences, Karachi, Pakistan; e Department of Medicine, Allama Iqbal Medical College, Lahore, Pakistan; f Department of Surgery, All India Institute of Medical Sciences, Bhubaneswar, India; g Department of Nursing, College of Nursing and Health Sciences, Jazan University, Jizan, Saudi Arabia; h Nursing Department, Hamad Medical Corporation, Doha, Qatar; i Department of Surgery, Federal Medical and Dental College, Islamabad, Pakistan; j Department of Medicine, Manipal College of Medical Sciences, Pokhara, Nepal

**Keywords:** carbohydrate loading, cholecystectomy, laparoscopy, patient outcomes, post-operative

## Abstract

**Background:**

Cholecystectomy is one of the most common procedures performed across the world. With efforts to maximize good postoperative status, one such technique involves preoperative carbohydrate loading.

**Methods:**

A thorough literature search was performed across three databases to identify articles published up to August 2024. The screening was conducted to select all primary studies evaluating the role of preoperative carbohydrate loading in patients undergoing laparoscopic cholecystectomy. Statistical analysis was conducted via RevMan using a random-effects model.

**Results:**

Thirteen records were included for quantitative synthesis, encompassing 827 participants (415 in the carbohydrate arm and 412 in the fasting arm). Carbohydrate loading was associated with significantly higher insulin levels compared to the control group (standard mean deviation [SMD]: −0.60; 95% confidence interval [CI]: −0.87, −0.34; *P* < 0.00001). However, other parameters like blood glucose (SMD: −0.19; 95%CI: −0.52, 0.14; *P* = 0.26) and homeostatic model assessment for insulin resistance (HOMA-IR) levels (SMD: −0.26; 95%CI: −0.82, 0.31; *P* = 0.37) had non-significant differences between both groups. On the contrary, post-operative pain levels were found to be lower among the carbohydrate group (SMD: −0.76; 95% CI: −1.35, −0.16; *P* = 0.01). Other outcomes like quality of recovery, use of antiemetics, or blood loss during operation had no significant differences between both groups.

**Conclusion:**

Our analysis suggests that preoperative carbohydrate loading is linked with reduced postoperative pain, incidence of nausea, and superior insulin parameters. Further research is needed to strengthen these findings.

**HIGHLIGHTS**

- Carbohydrate loading increases insulin levels (*P* < 0.01) postoperatively compared to fasting.
- It is associated with lesser postoperative complications like nausea and pain (*P* = 0.01)
- Carbohydrate loading must be explored for integration into ERAS protocols.

## Introduction

Cholecystectomy, which entails the extraction of the gallbladder, persists as one of the most frequently executed surgical interventions for addressing cholelithiasis, despite the emergence of non-invasive treatments, with nearly 300 000 surgical procedures being conducted annually in the United States[1]. The burden of gallstone diseases varies across different regions, and consequently the prevalence of cholecystectomy. Nevertheless, it is one of the most commonly performed procedures, with a lifetime prevalence of nearly 4% with a greater predilection toward women[2]. A study performed in South Africa highlights the increase in the number of cholecystectomies performed, denoting successful accessibility of this resource across the world[3]. Since the advent of cholecystectomy, surgeons have endeavored to make gallbladder operations less invasive by shifting from conventional open access to laparoscopic or transluminal techniques. This transition has led to shorter recovery periods and hospital stays for patients. Furthermore, these minimally invasive procedures offer cosmetic benefits, as the postoperative scar, such as the 4 cm scar from a mini-access cholecystectomy, is noticeably smaller[4]. Since 1992, laparoscopic cholecystectomy has been widely regarded as the preferred treatment option for individuals suffering from symptomatic cholelithiasis. Laparoscopic cholecystectomy constitutes 96% of these procedures[5]. Nevertheless, there exist multiple factors which increase the risk of adverse events in laparoscopic cholecystectomies. Older age, morbid obesity, and the severity of the disease play an important role in determining patient outcomes[6].

One of the various techniques introduced to improve postoperative outcomes is the concept of preoperative food intake. In the earlier times, it was believed that preoperative fasting minimizes reflux and aspiration risks during anesthesia, with patients typically abstaining from solids for 6–8 hours and liquids for 4 hours before surgery[7]. However, emerging evidence has highlighted the various pitfalls of this practice. Studies have described an increased risk of hyperglycemia and associated metabolic disturbances, higher anxiety and fatigue, and a higher risk of postoperative delirium.^[8-11]^ To alleviate this, the use of preoperative oral carbohydrates was introduced. By increasing insulin levels preoperatively, an anabolic state is maintained, reducing the risk of insulin resistance and indirectly inhibiting pro-inflammatory cytokines[12]. The use of preoperative oral carbohydrates may provide superior relief from hunger, pain, and postoperative insulin resistance compared to overnight fasting or a placebo for patients undergoing laparoscopic cholecystectomy[13]. Multiple emerging trials have tested the efficacy of preoperative glucose loading vis-a-vis fasting in laparoscopic cholecystectomies. However, certain studies also highlight the adverse events associated with carbohydrate loading; these include hyperglycemia among diabetic patients[12]. Thus, a gap in literature is whether carbohydrate loading truly benefits patients with respect to overall outcomes, while considering its specific adverse events.

We aim to collate these findings to objectively analyze the benefits of glucose loading, and whether it truly improves patient outcomes[14]. This report features carbohydrate-based drinks given at least 2 hours preoperatively, ranging from concentrations of 12.5 grams up to 67.0 grams. The results of this systematic review and meta-analysis can further help clinicians introduce this practice in their routine clinical setting. This study aims to synthesize data from all relevant clinical trials to rigorously assess the impact of preoperative oral carbohydrates on alleviating hunger, pain, and postoperative insulin resistance in comparison to overnight fasting for patients undergoing laparoscopic cholecystectomy.

## Methods

### Data sources and search strategy

This systematic review is in accordance with “Preferred Reporting Items for Systematic Review and Meta-Analysis” (PRISMA) and AMSTAR-2 (assessing the methodological quality of systematic reviews) guidelines[15]. Systematic search was done on three major databases, i.e., PubMed/MEDLINE, Cochrane Library, and Scopus from inception to 1 August 2024. References of relevant studies were also searched for potential studies. Search strategies employed for different databases are available in Supplementary Table 1 (available at: http://links.lww.com/MS9/A831). The review was prospectively registered in PROSPERO (CRD42024568960) before the initiation of the project.

### Study selection, eligibility criteria, and data extraction

All studies after systematic search were exported to Rayyan[16] for the removal of duplicates. Two investigators (M.S.Q. and M.O.L.) performed a screening of the article through title and abstracts, followed by a full-text review. The third reviewer (M.K.) was invited to resolve any discrepancies between the two reviewers.

The inclusion criteria included (i) studies reporting patients undergoing laparoscopic cholecystectomy, (ii) studies involving patients undergoing preoperative carbohydrate loading, (iii) studies reporting blood glucose or insulin levels post-operatively, that is, primary outcome, or studies reporting incidence of adverse events, postoperative nausea, and vomiting, pain, or other serum parameters, that is, secondary outcomes, and (iv) published randomized or non-randomized controlled trials. Exclusion criteria included (i) studies involving other surgical interventions than laparoscopic cholecystectomy and (ii) case reports, letters, and review articles are also excluded. No language-related restrictions were employed.

Data extraction was performed by two investigators (K.Z. and A.K.). The following data were extracted from the included studies: (i) baseline characteristics of included population, (ii) antiemetic use, (iii) blood loss during operation, (iv) cortisol level, (v) glucose level, (vi) insulin resistance via homeostatic model assessment for insulin resistance (HOMA-IR), (vii) hunger, (viii) insulin level, (ix) lactate level, (x) nausea, (xi) pain, (xii) pyruvate level, (xiii) quality of recovery, (xiv) triglycerides level, (xv) thirst, and (xvi) vomiting.

### Risk of bias assessment

The quality assessment of the included studies was conducted by two independent investigators (M.S.Q. and K.Z). Observational studies were evaluated using the Newcastle-Ottawa Scale (NOS)[17], while the Cochrane Risk of Bias Tool for Randomized Controlled Trials (RoB-2)[18] was utilized for assessing the quality of randomized controlled trials.

The NOS comprises three categories, each with a maximum score of 9. Studies scoring seven or more were classified as “good” quality, those with scores between two and six were deemed “fair” quality, and studies scoring one were categorized as “poor” quality.

For randomized controlled trials, quality was evaluated across the following domains: (i) randomization process, (ii) deviations from the intended interventions, (iii) missing outcome data, (iv) outcome measurement, and (v) selection bias in reported results. In instances of disagreement between the two independent reviewers, a third reviewer (M.O.L.) was consulted to achieve consensus.

### Statistical analysis

Data for this meta-analysis were analyzed using Review Manager (RevMan v5.4.1). Risk ratios (RRs) along with their corresponding 95% confidence intervals (CIs) were used for outcomes with dichotomous data, utilizing the Mantel–Haenszel method. Standard mean differences (SMD) were used due to differences in measurement scales. SMD was calculated using the inverse-variance method for continuous outcomes. A random-effects model was employed for data synthesis, and a *P* value of 0.05 or lower was considered significant in all analyses. Statistical heterogeneity within studies was estimated using Higgins I^2^ statistics[19] with values <50%, 55-75%, and >75% representing low, moderate, and high degrees of heterogeneity, respectively. A sensitivity analysis utilizing the leave-one-out method was conducted for outcomes with moderate to high heterogeneity.

## Results

### Literature search

**Figure 1.**
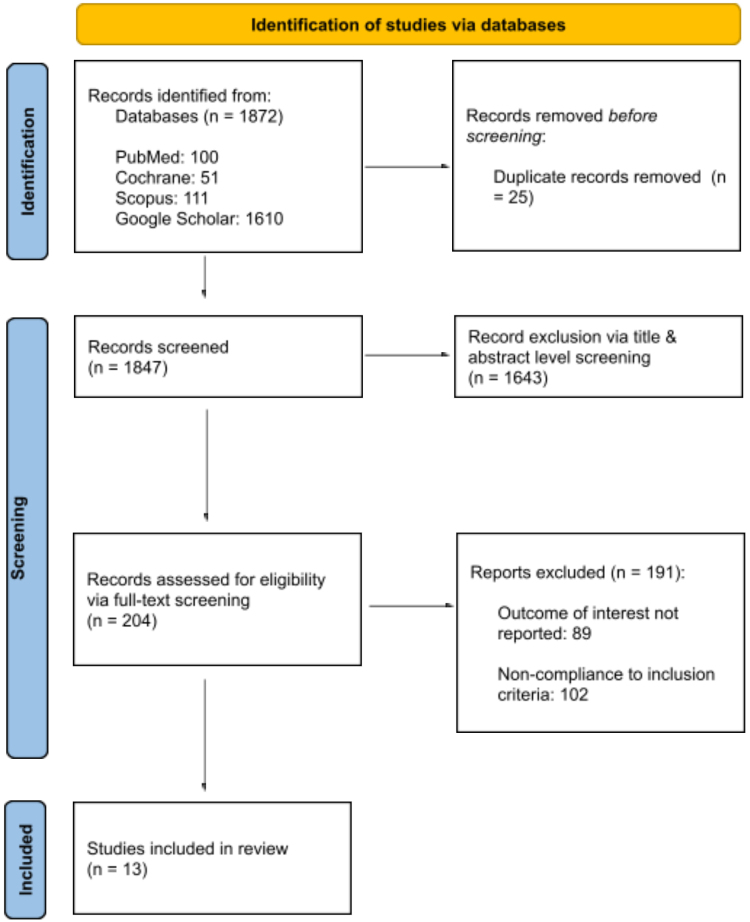
PRISMA flowchart of included studies.

The initial literature search yielded 1872 results. Out of these, 25 duplicates were removed. The studies were thoroughly reviewed based on abstracts and titles and additional 1643 studies were removed based on pre-defined inclusion and exclusion criteria. Finally, thirteen^[14,20-31]^ studies met our criteria and were included in the study as shown in the PRISMA flow chart (Fig. 1).

### Study characteristics and risk of bias assessment

The study included a total of 827 participants, with 415 in the preoperative carbohydrate loading intervention group and 412 in the fasting control group. The mean age of participants ranged between 35 and 53 years. The patient’s baseline characteristics are demonstrated in Table 1.

**Table 1:** Baseline table for included studies

		Participants			Mean age					Surgery duration (min)			ASA score			BMI	
Author	Country	CHO group	Fasting group	Total	CHO	Fasting	Volume administered	Time pre-op	Nutrient type	CHO	Fasting	ASA class	CHO	Fasting	Anesthesia administered	CHO	Fasting
Wang 2024	China	42	45	87	49.0 ± 13.0	49.0 ± 12.3	200 ml	2–4 hr before surgery	12.5 g carbohydrate	57.0 ± 15.5	58.0 ± 17.6	1–2	32/10	36/9	Midazolam, etomidate, sufentanil, rocuronium	25.1 ± 2.7	24.7 ± 3.1
Balabolu 2023	India	50	50	100	47.26 ± 13.06	46.84 ± 12.02	200 ml	2 hr	25 g of carbohydrate	-	-	1–2	35/15	39/11	Propofol and vecuronium	22.49 ± 2.21	20.64 ± 2.47
Mousavie 2021	Iran	26	26	52	38 ± 11	41 ± 13	200 ml	2 hr	25 g dextrose	132 ± 36	108 ± 0.5	1–2	13/13	18/8	Propofol and atracurium	26.8 ± 4	28 ± 5
Helminen 2019	Finland	57	56	113	47 ± 13	46 ± 11	200 ml	2 hr	67 g carbohydrate 8 g protein	59 ± 23	68 ± 25	1–2	33/24	39/17	Fentanyl, propofol and a muscle relaxant.	29 ± 5	28 ± 4
Onalan 2019	Turkey	25	25	50	53 (16)	54 (14)	800 ml + 400 ml	8 hr + 2 hr	12.5% glucose	77.37 ± 21.50	78.25 ± 25.97	1–2	-	-	Propofol, fentanyl and rocuronium	28.3 ± 3.7	29 ± 3.3
Lee 2018	Korea	46	44	90	50 ± 13	48 ± 12	400 ml + 400 ml	Morning + 2 hr	Carbohydrate	52 ± 20	44 ± 20	1–2	-	-	Propofol, remifentanil, rocuronium	24.5 ± 4.8	25.4 ± 3.8
Pędziwiatr 2015	Poland	20	20	40	53.7 ± 15.29	55 ± 9.55	400 ml	2 hr	Carbohydrate	78.25 ± 25.97	77.37 ± 21.50	1,2,3	6/13/1	3/14/3	-	28.78 ± 4.85	28.76 ± 5.31
Ravanini 2015	Brazil	21	17	38	47.7 ± 14.5	41 ± 14.2	200 ml	2 hr	33.5% carbohydrate and 4% protein	91	74	1–2	12/9	9/8	Dipyrone	-	-
Singh 2015	India	40	40	80	43.2 ± 15.85	44.40 ± 11.45	400 ml + 200 ml	Evening + 2 hr	12.5 carbohydrate	-	-	-	-	-	-	-	-
DOCK-NASCIMENTO 2012	Brazil	10	9	19	-	-	400 ml + 200 ml	8 hr + 2 hr	12.5% maltodextrin	-	-	1–2	-	-	Alfentanil	-	-
Dock-Nascimento 2012	Brazil	12	12	24	35 ± 4.1	40 ± 3.3	400 ml + 200 ml	8 hr + 2 hr	Maltodextrin and water	50 ± 5	66 ± 8	1–2	10/2	8/4	General anesthesia and cefazolin	25.8 ± 0.6	26.1 ± 1.4
Faria 2009	Brazil	11	10	21	45.01 ± 14.44	47.58 ± 11.64	200 ml	2 hr	12.5% maltodextrin	117.99 ± 23.54	114.67 ± 25.86	1–2	5/6	4/6	General anesthesia and cefazolin	-	-
Hausel 2005	Sweden	55	58	113	48 · 3 ± 14 · 6	48 · 0 ± 14 · 9	800 + 400 ml	Evening + 2 hr	12.5 carbohydrate	69 ± 36	75 ± 41	1–2	-	-	General anesthesia and thiopental	24 · 2 ± 3 · 0	25.2 ± 2.8

Quality assessment was conducted using the Cochrane Risk of Bias Tool for the randomized controlled trials (RCTs). Among the thirteen included studies, two were deemed to have having “low” risk of bias^[14,31]^, two were found to have “some concerns”^[23,25]^, and the remaining nine studies were judged to have a “high” risk of bias. Bias resulting from measurement of outcome,^[22,24,26-30]^ missing outcomes data[20], and deviation from intervention^[21,22]^ contributed to high bias (Supplementary Figure 1, available at: http://links.lww.com/MS9/A831; Supplementary Figure 2, available at: http://links.lww.com/MS9/A831).

### Results of meta-analysis

#### Glucose and insulin profiles

**Figure 2.**
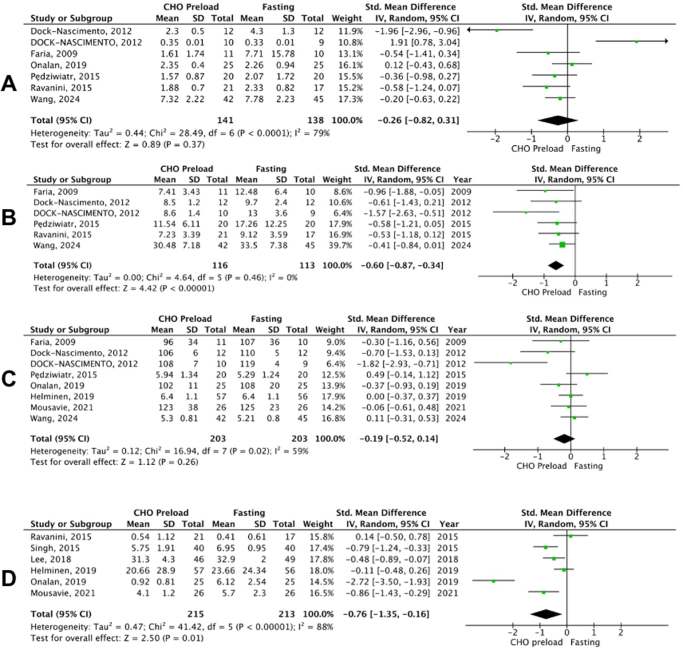
Forest plot of (A) HOMA-IR, (B) insulin levels, (C) glucose levels, and (D) pain after administration of carbohydrate loading versus control.

Seven studies out of 13 reported HOMA-IR A statistically non-significant difference between the two groups was observed (SMD: −0.26; 95% CI: −0.82 to 0.31; *P* = 0.37; I^2^ = 79%; Fig. 2). However, the heterogeneity was high (I^2^ = 79%). To tackle the high heterogeneity among studies, sensitivity analysis was attempted. However, it failed to identify the source of heterogeneity. Furthermore, publication bias was assessed, revealing no significant heterogeneity (*P* = 0.18; Supplementary Figure 3, available at: http://links.lww.com/MS9/A831).

Insulin level was reported by six studies. A significant difference was observed between the two groups in the analysis with the insulin levels favoring the carbohydrate loading group (SMD: −0.60; 95% CI: −0.87 to −0.34; *P* < 0.00001; Fig. 2).

The data from eight studies were analyzed for blood glucose level and it showed no significant difference between the two groups (SMD: −0.19; 95% CI: −0.52 to 0.14; *P* = 0.26; I^2^ = 59%; Fig. 2). Significant heterogeneity (I^2^ = 59%) was found. Sensitivity analysis was performed and removing DOCK-NASCIMENTO (2012), which was the study with high risk of bias, brought heterogeneity down to an acceptable 19%, without changing the statistical significance of the overall result (SMD: −0.05; 95% CI: −0.28 to 0.18; *P* = 0.68; I^2^ = 19%; Supplementary Figure 4, available at: http://links.lww.com/MS9/A831). Furthermore, publication bias was assessed, revealing significant heterogeneity (*P* = 0.02; Supplementary Figure 5, available at: http://links.lww.com/MS9/A831).

#### Pain

Pain was reported in six of the studies, and a significant difference was noted between the two groups, favoring the carbohydrate loading group (SMD: −0.76; 95% CI: −1.35 to −0.16; *P* = 0.01; I^2^ = 88%; Fig. 2). High heterogeneity was observed (I^2^ = 88%). To tackle the high heterogeneity among studies, sensitivity analysis was attempted. However, it failed to identify the source of heterogeneity. Furthermore, publication bias was assessed, revealing no significant heterogeneity (*P* = 0.12; Supplementary Figure 6, available at: http://links.lww.com/MS9/A831).

#### Quality of recovery

**Figure 3.**
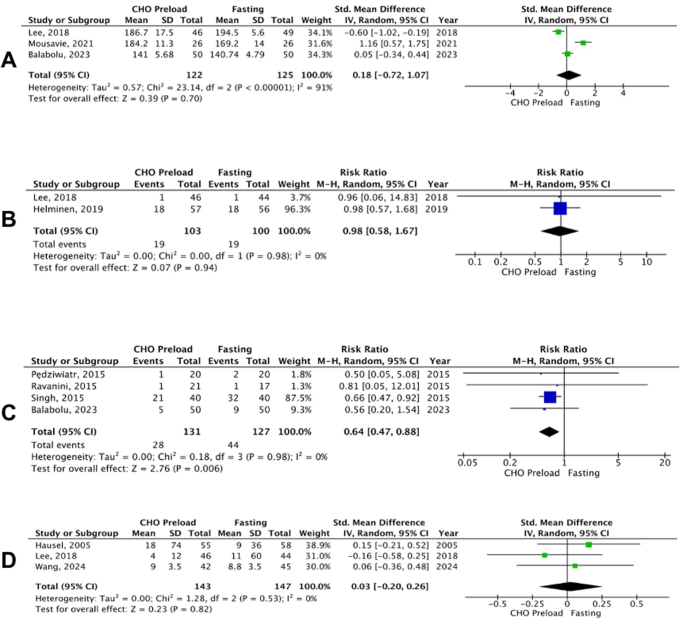
Forest plot of (A) quality of recovery, (B) antiemetic use, (C) nausea, and (D) blood loss after administration of carbohydrate loading versus control.

Quality of recovery was reported by three studies. The difference between the carbohydrate loading group and fasting group was not significant (SMD: 0.18; 95% CI: −0.72 to 1.07; *P* = 0.70; I^2^ = 91%; Fig. 3). High heterogeneity was observed; however, sensitivity analysis failed to find the source of heterogeneity.

#### Use of antiemetics

Antiemetic use was reported in two of the studies. No significant difference was observed between the carbohydrate loading group and fasting group in terms of antiemetic use (RR: 0.98; 95% CI: 0.58 to 1.67; *P* = 0.94; Fig. 3).

#### Incidence of nausea

Nausea was reported as an outcome by four studies and a significant difference was observed with risk ratio more toward the carbohydrate loading group (RR: 0.64; 95% CI: 0.47 to 0.88; *P* = 0.006; Fig. 3).

#### Blood loss during operation

This outcome was reported by three studies. The difference in blood loss during operation between the two groups was insignificant (SMD: 0.03; 95% CI: −0.20 to 0.26; *P* = 0.82; Fig. 3).

#### Lactate levels

**Figure 4.**
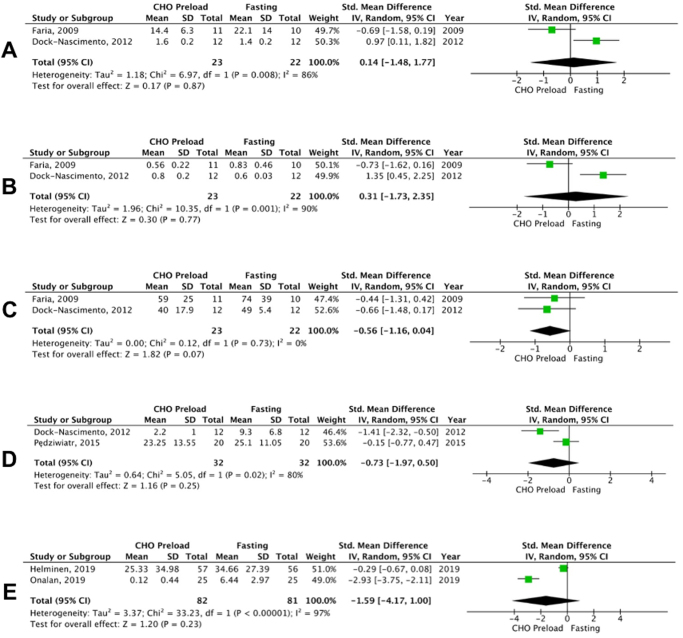
Forest plot of (A) lactate level, (B) pyruvate level, (C) triglycerides, (D) cortisol level, and (E) hunger after administration of carbohydrate loading versus control.

Only two studies reported the lactate levels and no significant difference was observed between the carbohydrate loading group and the fasting group (SMD: 0.14; 95% CI: −1.48 to 1.77; *P* = 0.87; I^2^ = 86%; Fig. 4). High heterogeneity was observed (I^2^ = 86%); however, sensitivity analysis could not be performed due to a limited number of studies.

#### Pyruvate levels

Pyruvate levels were reported by two studies and no significant difference was observed between the carbohydrate loading group and fasting group (SMD: 0.31; 95% CI: −1.73 to 2.35; *P* = 0.77; I^2^ = 90%; Fig. 4). Sensitivity analysis could not be performed due to the limited number of studies.

#### Triglyceride levels

This outcome was reported by 2 out of 13 studies and the difference between the two groups was found to be insignificant (SMD: −0.56; 95% CI: −1.16 to 0.04; *P* = 0.07; Fig. 4). Sensitivity analysis could not be performed due to the limited number of studies.

#### Cortisol levels

Out of 13 studies, 2 of them reported cortisol levels. No significant difference in cortisol levels was observed between the carbohydrate preload group and the fasting group (SMD: −0.73; 95% CI: −1.97 to 0.50; *P* = 0.25; I^2^ = 80%; Fig. 4). High heterogeneity was observed (I^2^ = 80%); however, due to a limited number of studies sensitivity analysis could not be performed.

#### Hunger

Hunger was reported as an outcome by only two studies and no significant difference was observed between the two groups (SMD: −1.59; 95% CI: −4.17 to 1.00; *P* = 0.23; I^2^ = 97%; Fig. 4). A high level of heterogeneity was noted (I^2^ = 97%); however, sensitivity analysis could not be conducted due to the limited number of studies.

## Discussion

The influence of carbohydrate intake before surgery on outcomes following laparoscopic cholecystectomy has been increasingly noted. Our study revealed that HOMA-IR and glucose levels were not statistically significant between the preoperative carbohydrate loading and fasting groups, with sensitivity analysis unable to resolve the high heterogeneity. Insulin levels, however, were significantly lower in the carbohydrate loading group. Glucose levels were also not statistically significant between groups. Pain was significantly lower in the carbohydrate loading group, though heterogeneity remained unresolved. No significant differences were observed for quality of recovery, antiemetic use, blood loss, lactate, pyruvate, triglycerides, cortisol levels, or hunger, with heterogeneity frequently high but sensitivity analyses unable to identify clear sources. Nausea was significantly reduced in the carbohydrate loading group.

Preoperative carbohydrate loading has been shown to mitigate the stress response associated with surgery, which is particularly relevant in laparoscopic cholecystectomy. Yuan *et al* reported that oral enzyme-digested rice flour solutions administered preoperatively improved gastric emptying and reduced insulin resistance in patients undergoing laparoscopic cholecystectomy, suggesting that carbohydrate drinks can enhance postoperative recovery by modulating metabolic responses[32]. This is in concordance with our study, where the carbohydrate preload routine was found to significantly increase the insulin levels implying a probable mechanism through which carbohydrate loading may benefit postoperative results. Yadav *et al* also complement the results of our study showing that preoperative carbohydrate loading could decrease the insulin resistance of patients undergoing laparoscopic cholecystectomy especially those with diabetes[33]. This is also similar to a study conducted by Noba *et al* which showed that preoperative carbohydrate drinks reduced insulin resistance[34].

Qi *et al* noted that there is a possibility of decreasing postoperative insulin resistance by giving carbohydrates before surgery by lowering stress hormone levels and altering insulin receptor kinetics[35]. This is especially important to our study on laparoscopic cholecystectomy, as improvement of insulin resistance could potentially improve recovery profiles. Moreover, Yang *et al* also showed that preoperative carbohydrate loading could improve insulin sensitivity which is vital during the postoperative period[36]. These enhancements unravel the underlying physiological mechanisms that are prevalent for these improvements and are rooted within the modulation of definite metabolic pathways. It was believed that preoperative carbohydrate loading reduces pyruvate dehydrogenase kinase 4 (PDK4), which contributes to insulin resistance and increases phosphoinositide-3-kinase/protein kinase B (PI3K/PKB) signal transduction, which is important for insulin activity[37]. This biochemical response not only helps in reducing insulin resistance but also in better metabolism of glucose which is very much required during the postoperative period.

Perioperative nausea and vomiting (PONV) is a common adverse effect after any surgical intervention, which affects the patient’s quality of life. In line with the above-mentioned finding, Tavalaee *et al* reported that administering CHO loading before surgery reduced the occurrence of PONV in laparoscopic cholecystectomy patients[38]. Their study included a total of 120 patients, suggesting that carbohydrate drinks can modulate metabolic fluctuations and thereby prevent nausea. Our results also showed that pain was significantly lower in the carbohydrate loading group which was in line with the results of a previous study which concluded that carbohydrate loading could reduce postoperative pain in patients who had laparoscopic cholecystectomy[38]. In the same respect, Baz *et al* also showed that intraperitoneal levobupivacaine for routine postoperative pain relief was safe and effective in reducing pain scores following laparoscopic cholecystectomy suggesting that multimodal analgesia strategies, including carbohydrate loading, could enhance pain management[39].

While carbohydrate loading can be administered in different forms, the most popular and accepted formulation worldwide is the use of carbohydrate drink, comprising a solution of 800 ml carbohydrate in the evening prior to surgery, and then 400 ml 2–3 hours before surgery[40]. Maltodextrin the major component of this solution, which is cleared from the gastrointestinal system by 2 hours, therefore does not pose a risk for aspiration pneumonia[41].

The decreased postoperative pain following carbohydrate loading can be explained by the modulation of inflammatory and metabolic indices. Studies have demonstrated that carbohydrate-containing drinks reduce stress hormone levels, which are known to increase pain sensation[42]. However, since carbohydrate loading improves the overall metabolic state, it may also be useful in maintaining muscle integrity and strength, which has been directly linked to reductions in post-surgery pain[43]. Furthermore, our meta-analysis conclusions are in accordance with Rizvanović *et al*, where preoperative carbohydrate loading was identified to have the potential to reduce postoperative complications, as well as enhance recovery indexes in patients with colorectal surgery, which shares similar physiological stress response to laparoscopic cholecystectomy[44]. This suggests that the benefits of carbohydrate loading may extend beyond specific surgical procedures. In terms of quality of recovery, carbohydrate loading has demonstrated benefits for postoperative outcomes by stabilizing metabolic processes. By providing a readily available source of glucose, carbohydrate loading helps maintain metabolic homeostasis, which is essential for recovery. Patients who undergo carbohydrate loading report higher quality of recovery scores, likely due to the reduced incidence of postoperative complications and improved overall well-being[45].

The implications of our findings also extend to clinical practice, particularly in the context of Enhanced Recovery After Surgery (ERAS) protocols. The ERAS guidelines recommend the use of carbohydrate (CHO) preloading to promote postoperative recovery, shorten hospital stays, and increase patient satisfaction[46]. The psychological impact of preoperative carbohydrate loading should not be overlooked. Patients who consume carbohydrates before surgery often report feeling more comfortable and less anxious, which can contribute to lower pain perception. The alleviation of anxiety may be linked to increased serotonin levels, as suggested by research on the effects of carbohydrate intake on mood[47]. There may be several barriers to the implementation of ERAS protocols, specifically carbohydrate loading, into the clinical setting. Firstly, the lack of standardized ERAS-oriented protocols in hospitals across the world lead to inconsistent or absent guidelines for preoperative carbohydrate loading, affecting the widespread usage of this technique. Additionally, there are significant cost-related concerns with the usage of carbohydrate loading, especially among the low-income or underdeveloped regions whose patients may not be able to afford the extra costs associated with this feature, thus limiting the routine usage among such hospitals. Furthermore, there are various unexplored patient-specific concerns, especially among those individuals who have underlying metabolic disorders (i.e., diabetes mellitus), demanding further research prior to the widespread use of carbohydrate loading. In order to curb these barriers, it is important to promote education and awareness among institutions, encourage the integration of standardized protocols, and explore various other cost-effective solutions for carbohydrate drinks that would serve as excellent alternatives within resource-limited areas.

Although our meta-analytical findings provide pertinent clinical insights into one of the most common surgical procedures within the field of general surgery, evaluation of other subspecialties and procedures are necessitated. Laparoscopic cholecystectomy is frequently considered to be a minimally invasive procedure with a low rate of complications, permitting a quicker hospital discharge. In contrast, other complex surgeries face a greater incidence of adverse effects and subsequently require additional consideration in terms of preoperative carbohydrate loading and postoperative adverse effects. Hence, further original clinical research into other surgical procedures (i.e., head and neck, orthopedic, and cardiothoracic surgery) is mandated, as our preliminary literature search failed to reveal sufficient studies for a purposeful meta-analysis on other techniques.

Considerable heterogeneity was observed within our meta-analysis. Mainly in terms of post-operative outcomes, high variability was noted in HOMA-IR levels, pain and other patient-dependent outcomes. Considerable variation was also noted in lab parameters. A main contribution to this variability is the difference in baseline parameters of the patients. Presence of any comorbidity, including diabetes, holds an adverse effect on the post-operative recovery of patients. Additionally, medications taken by the patient preoperatively and intra-operatively significantly influence outcomes of patients, especially in those on chronic steroid use. To account for these factors, future studies must adopt a randomized controlled design, allowing comparability between cohorts, including presence of multimorbidity pre-procedure.

Our study has certain limitations. Firstly, the sensitivity analysis could not identify the source of heterogeneity, and the limited number of studies prevented an assessment of publication bias. Additionally, there were clear inconsistencies in the concentration, timing of administration, and volume of carbohydrate across the studies. Second, personal experiences of discomfort were likely shaped by factors such as cultural background, communication skills, and individual perspectives. Third, inherent differences related to race, gender, participant demographics, and the use of non-standardized insulin measurement methods without an established universal threshold also posed challenges. Therefore, a thorough assessment of the study population and the insulin measurement techniques used is essential for accurate interpretation. Additionally, various other unknown confounding factors may limit the strength of our results, including differences in anesthesia protocols, varying metabolic profiles, and disparate procedural parameters based on operator preferences. Finally, a severely high risk of bias was identified within the included studies, with 9 of the 13 studies reporting a “high” risk of bias. This may potentially threaten the validity of the results by increasing the heterogeneity observed within the studies. Further studies with robust and transparent methodologies are encouraged.

## Conclusion

In conclusion, preoperative carbohydrate loading shows promise in improving postoperative outcomes for patients undergoing laparoscopic cholecystectomy. Our findings indicate that carbohydrate intake can reduce insulin resistance, lower pain levels, and decrease nausea and vomiting. These benefits may enhance recovery quality and patient satisfaction. However, limitations such as high heterogeneity warrant further investigation to standardize carbohydrate protocols and explore the underlying physiological mechanisms. Implementing carbohydrate loading may be a valuable strategy for optimizing surgical recovery.

## Data Availability

All data generated have been either published in the manuscript or available in the supplementary file.

## Research registration unique identifying number (UIN)

Prospectively registered via PROSPERO with identification number of CRD42024568960.
